# Novel Snail1 Target Proteins in Human Colon Cancer Identified by Proteomic Analysis

**DOI:** 10.1371/journal.pone.0010221

**Published:** 2010-04-20

**Authors:** María Jesús Larriba, Juan Casado-Vela, Natalia Pendás-Franco, Raúl Peña, Antonio García de Herreros, María Teresa Berciano, Miguel Lafarga, J. Ignacio Casal, Alberto Muñoz

**Affiliations:** 1 Instituto de Investigaciones Biomédicas “Alberto Sols”, Consejo Superior de Investigaciones Científicas-Universidad Autónoma de Madrid, Madrid, Spain; 2 Centro Nacional de Biotecnología, Consejo Superior de Investigaciones Científicas, Madrid, Spain; 3 Institut Municipal d'Investigació Mèdica (IMIM)-Hospital del Mar, Barcelona, Spain; 4 Departamento de Anatomía y Biología Celular, Instituto de Formación e Investigación Marqués de Valdecilla, Universidad de Cantabria, Santander, Spain; 5 Centro de Investigaciones Biológicas, Consejo Superior de Investigaciones Científicas, Madrid, Spain; Centre de Regulació Genòmica, Spain

## Abstract

**Background:**

The transcription factor Snail1 induces epithelial-to-mesenchymal transition (EMT), a process responsible for the acquisition of invasiveness during tumorigenesis. Several transcriptomic studies have reported Snail1-regulated genes in different cell types, many of them involved in cell adhesion. However, only a few studies have used proteomics as a tool for the characterization of proteins mediating EMT.

**Methodology/Principal Findings:**

We identified by proteomic analysis using 2D-DIGE electrophoresis combined with MALDI-TOF-TOF and ESI-linear ion trap mass spectrometry a number of proteins with variable functions whose expression is modulated by Snail1 in SW480-ADH human colon cancer cells. Validation was performed by Western blot and immunofluorescence analyses. Snail1 repressed several members of the 14-3-3 family of phosphoserine/phosphothreonine binding proteins and also the expression of the Proliferation-associated protein 2G4 (PA2G4) that was mainly localized at the nuclear Cajal bodies. In contrast, the expression of two proteins involved in RNA processing, the Cleavage and polyadenylation specificity factor subunit 6 (CPSF6) and the Splicing factor proline/glutamine-rich (SFPQ), was higher in Snail1-expressing cells than in controls. The regulation of 14-3-3ε, 14-3-3τ, 14-3-3ζ and PA2G4 by Snail1 was reproduced in HT29 colon cancer cells. In addition, we found an inverse correlation between 14-3-3σ and Snail1 expression in human colorectal tumors.

**Conclusions/Significance:**

We have identified a set of novel Snail1 target proteins in colon cancer that expand the cellular processes affected by Snail1 and thus its relevance for cell function and phenotype.

## Introduction

The transcription factor Snail1 regulates the biology of fibroblasts and its upregulation in epithelial cells causes a change to a mesenchymatic phenotype, a process known as epithelial-to-mesenchymal transition (EMT). This is a transdifferentiation shift in which epithelial cells lose adhesiveness and polarity and acquire the spindle morphology and migratory capacity characteristic of fibroblasts as a result of a drastic alteration in the gene expression profile [1], [2]. EMT plays important physiological roles during embryogenesis and wound healing, and is also crucial for the acquisition of tumoral invasiveness, the initial step of the metastatic cascade in cancer [1], [2].

Snail1 appears to have a master role in EMT, although several other factors such as Snail2 (Slug), Zeb1 (δEF1), Zeb2 (Sip1), Twist1, E47 and E2-2 are also known to induce or contribute to the EMT in different types of cells [2], [3]. These factors promote partially different gene expression profiles that have in common the repression of genes encoding adhesion proteins (E-cadherin, Occludin and several claudins) and the induction of mesenchymal markers such as Vimentin, Fibronectin, and proteases and factors that promote cell movement [3], [4]. Snail1 was the first characterized repressor of the invasion suppressor gene *CDH1* that encodes for the crucial adhesion protein E-cadherin [5], [6]. In addition, Snail1 has recently been shown to activate Wnt/β-Catenin signaling and Nuclear factor *kappa* B activity [7], [8], and it abrogates the inhibition of Wnt/β-Catenin pathway caused by the antitumoral compound 1α,25-dihydroxyvitamin D_3_ [9].

Snail1 is upregulated in several human cancers and is frequently associated with invasiveness, metastases and poor prognosis [3]. Snail1 RNA is undetectable in normal colon mucosa but it becomes upregulated in 60–70% of colon cancers [10]–[12]. A recent study has shown that Snail1 protein is expressed in 79% of colon tumors, usually in the tumor-stroma interphase. Snail1 is found in both the epithelial tumor tissue and the adjacent stroma in 56% of colon tumors, whereas in 21% of the cases it is only present in stromal cells with fibroblastic phenotype [13].

A number of studies have focused on the transcriptomic analysis of the EMT promoted by Snail1 and other transcription factors, or by genes or agents such as oncogenic Ras or Transforming growth factor β that induced them [4], [14]–[17]. However, only a few studies have used proteomics as a tool for the characterization of proteins mediating EMT [18]. The majority of these studies have employed 2D-PAGE followed by MALDI-TOF mass spectrometry to identify proteins differentially-expressed in tumor tissues and/or cell lines [18]–[20]. A few studies have used iTRAQ technology followed by 2D-LC separation of peptides and mass spectrometry [18], [21], [22]. Here, we have used 2D-DIGE technology followed by MALDI-TOF-TOF and ESI-linear ion trap mass spectrometry. The combination of these techniques is a more sensitive and reliable approach for the identification of minor differences in protein expression between human colon cancer cells with low or high levels of Snail1. These techniques have given us the opportunity to take a deeper insight into the proteomic changes associated to Snail1 overexpression. In this study, we have identified a number of proteins previously unknown as Snail1 targets in human colon cancer cells. Some of them are involved in the control of cellular gene expression and phenotype, and are probable mediators of the tumorigenic action of Snail1.

## Results

**Figure 1:**
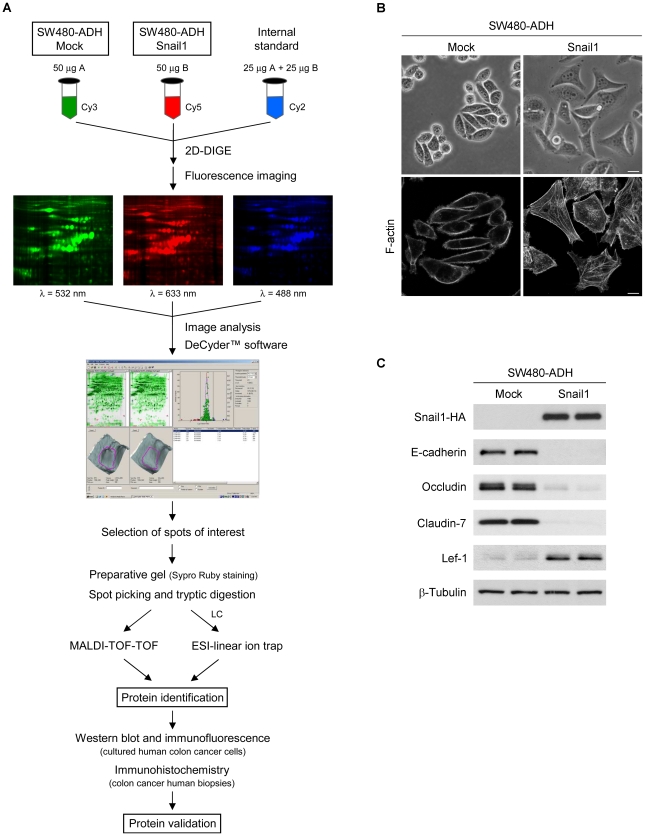
Experimental workflow and characterization of SW480-ADH Mock and Snail1 cells. (A) Scheme of the workflow followed in this study. (B) Representative phase-contrast micrographs (upper panels) and confocal laser immunofluorescence images showing F-actin staining (lower panels) of SW480-ADH Mock and Snail1 cells. Scale bar, 20 µm (upper panels) and 10 µm (lower panels). (C) Western blot analysis of whole-cell extracts from SW480-ADH Mock and Snail1 cells showing Snail1 overexpression and its effect on the expression of epithelial (E-cadherin, Occludin and Claudin-7) and mesenchymal (Lef-1) markers. β-Tubulin was used as a loading control.

Most genes previously characterized as regulated by Snail1 encode plasma membrane proteins involved in cell adhesion or cytoskeletal components. To widen our knowledge of Snail1 effects in human colon cancer, we employed an integrative strategy to identify nuclear proteins regulated by Snail1 in this neoplasia (Figure 1A). We used 2D-DIGE electrophoresis combined with MALDI-TOF-TOF and ESI-linear ion trap mass spectrometry to compare the pattern of proteins present in nuclear extracts from human colon cancer SW480-ADH cells expressing a retrovirally-transduced Snail1 cDNA (Snail1 cells) with that of mock-infected cells (Mock cells). A subset of the identified proteins was validated by Western blot and immunofluorescence analyses of human colon cancer cells, and by immunohistochemistry of human colon cancer biopsies. We have previously characterized the cellular model used in this study [7], [10] and found that Snail1 overexpression promoted the acquisition of a more stellate cell morphology (Figure 1B), the repression of the epithelial markers E-cadherin, Occludin and Claudin-7, and the induction of the mesenchymal protein Lef-1 (Figure 1C).

### Differential protein expression analysis of nuclear fractions from human colon cancer cells with high or low Snail1 expression

**Figure 2:**
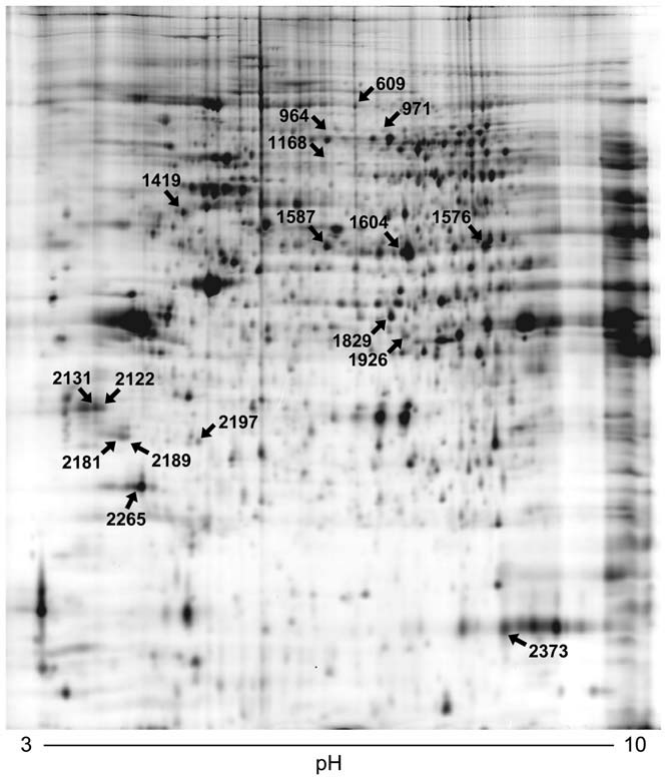
Differential protein expression analysis of SW480-ADH Snail1 and Mock cells by 2D-DIGE electrophoresis. Nuclear extracts from SW480-ADH Mock and Snail1 cells were differentially labeled with Cy3 and Cy5 dyes. An internal standard combining proteins from both extracts was included in all gels after labeling with Cy2 dye. 3–10 NL IPG strips were used for the IEF and standard SDS-PAGE for the second dimension. Representative 2D image shows the spot map corresponding to the internal standard, which is common to all gels analyzed. Spots indicated with arrows were informative for protein identification by mass spectrometry (see Table 1 for code assignment). Selected spots showed a statistical variance of volume ratios within the 95th confidence level (Student's *t*-test, *p*<0.05).

Nuclear extracts from SW480-ADH Mock and Snail1 cells were analyzed by 2D-DIGE as described in Materials and Methods. Protein pattern changes were quantitatively and statistically analyzed by using the DeCyder software v.6.5 considering the 12 spot maps included in the study. Figure 2 shows a representative 2D map of the nuclear SW480-ADH proteins. Each gel was analyzed individually to calculate the volume ratios between individual samples and the internal pool, which was used as the standard reference. Then, all the images were combined and analyzed together by using the BVA module. Few variations in protein abundance were observed between Mock and Snail1 cells. A paired *t*-test was used to select 22 spots whose expression variation between both cell types was statistically significant (Student's *t*-test, *p*<0.05). These spots were selected as spots of interest and annotated on the master gel (Figure 2). Protein identification was performed by two methods: (1) peptide mass fingerprinting using MALDI-TOF-TOF and (2) ESI-linear ion trap and MS/MS peptide sequencing (applying a restrictive false positive discovery rate (FDR) cut-off, FDR≤1%). In both cases, identification with more than one peptide was a requisite. By using this double approach, 19 proteins were identified in 17 out of 22 spots (Table 1). Only five proteins were identified by MALDI-TOF-TOF due to the low levels of expression. Eighteen proteins were identified by ESI-linear ion trap, with two spots containing more than one protein. In those cases, proteins were listed according to the number of identified peptides. Four proteins were identified by both techniques (Table 1).

**Table 1:** List of proteins differentially expressed in SW480-ADH Snail1 and Mock cells.

Spot No.	SwissProt Access. No.	Gene symbol	Protein name	MW (Da)	p*I*	Seq. cov. (%)/No. pept.^a^	Av. ratio^b^	Function
609	P35221	CTNNA1	Catenin alpha 1	100,071	5.95	2.54/2	1.59	Cell adhesion
964	P27986	PIK3R1	PI3K regulatory subunit alpha	83,598	5.84	4.97/3	2.31	PI3K cascade
971	Q05682	CALD1	Caldesmon	93,250	5.63	12.99/12	3.61	Actin binding
1168	Q16630	CPSF6	Cleavage and polyadenylation specificity factor subunit 6	9,210	6.66	4.39/2	1.99	mRNA processing
1419	P08670	VIM	Vimentin	53,652	5.06	27.25/21	1.66	Cytoskeleton
	P06576	ATP5B	Mitochondrial ATP synthase subunit beta	56,560	5.26	4.54/4		Proton transport
	Q15007	WTAP	Pre-mRNA splicing regulator WTAP	44,244	5.12	7.07/2		RNA splicing
1576	P06733	ENO1	Alpha enolase	47,169	7.37	16.59/10	−2.47	Transcription regulator
1587	O60884	DNAJA2	DnaJ homolog subfamily A member 2	45,746	6.06	4.85/2	−2.14	Protein binding
1604	Q9UQ80	PA2G4	Proliferation-associated protein 2G4	43,787	6.13	5.58/4	−1.51	Cell proliferation
1829	P07355	ANXA2	Annexin A2	38,604	7.57	3.20/2	−4.10	Phospholipase inhibitor
1926	Q14847	LASP1	LIM and SH3 domain protein 1	29,717	6.61	15.71/8	−2.44	Actin binding
	P31942	HNRNPH3	Heterogeneous nuclear ribonucleoprotein H3	36,926	6.37	10.12/5		RNA binding
2122	P62258	YWHAE	14-3-3 protein epsilon	29,174	4.63	14.12/3	−1.67	Cell signaling
2131	P62258	YWHAE	14-3-3 protein epsilon	29,174	4.63	9.41/2	−1.78	Cell signaling
2181	Q04917	YWHAH	14-3-3 protein eta	28,219	4.76	10.57/2	−2.26	Cell signaling
2189	P27348	YWHAQ	14-3-3 protein tau	27,764	4.68	5.30/4	−1.97	Cell signaling
2197	P43487	RANBP1	Ran-specific GTPase-activating protein	23,310	5.19	10.95/3	−1.88	Ran GTPase
2265	P13693	TPT1	Translationally-controlled tumor protein 1	19,595	4.84	32.00/68	−1.89	Apoptosis
2373	P62937	PPIA	Peptidyl-prolyl cis-trans isomerase A	18,012	7.68	7.88/2	−2.80	Protein folding

All the listed proteins showed a statistical difference of spot volume ratio between SW480-ADH Snail1 and Mock cells within the 95th confidence level (Student's *t*-test, *p*<0.05).

The majority of the proteins were identified only by ESI-linear ion trap, except of TPT1 that was identified by MALDI-TOF-TOF, and VIM, ENO1, PA2G4 and LASP1 that were identified using both techniques.

^a^Sequence coverage (%) and number of peptides identified with ≤1% FDR (false discovery rate cut-off against decoy-concatenated randomized database). Coverage and FDR were determined by SEQUEST algorithm for all proteins except for TPT1, which score was determined by MASCOT algorithm.

^b^Average ratio of protein expression between SW480-ADH Snail1 and Mock cells.

### Protein ontology analysis of identified proteins

Among the proteins altered by Snail1 overexpression we identified α-Catenin (CTNNA1), the PI3K regulatory subunit α (PIK3R1), Caldesmon (CALD1), the Cleavage and polyadenylation specificity factor subunit 6 (CPSF6), and Vimentin (VIM) as upregulated proteins; and Proliferation-associated protein 2G4 (PA2G4, also known as ErbB3-binding protein 1 or EBP1), Ran-specific GTPase-activated protein (RANBP1), and several members of the 14-3-3 protein family (YWHAE, YWHAH and YWHAQ) among the downregulated proteins (Table 1). Despite working with nuclear extracts, only 7 out of 19 proteins were assigned to the nuclear compartment (CPSF6, WTAP, ENO1, PA2G4, HNRNPH3, RANBP1 and PPIA), while the remaining were preferentially cytosolic or associated to the cytoskeleton. However, we cannot discard that the latter proteins were partially or temporarily present in the nuclear compartment. Regarding biological function, several proteins were involved in cell adhesion (CTNNA1) or components of the cytoskeleton (CALD1, VIM and LASP1). However, most of the proteins were implicated in signal transduction (PI3KR1, RANBP1 and the 14-3-3 family members) and RNA processing (CPSF6, WTAP and HNRNPH3) (Table 1). Some of these proteins were selected for validation. Given the high homology existing among the 14-3-3 proteins and between some of the identified peptides (i.e. –VISSIEQK– for 14-3-3τ and –VLSSIEQK– for 14-3-3σ, which makes them virtually indistinguishable), we decided to include all the members of the family in the validation analyses.

### Validation of candidate target proteins

**Figure 3:**
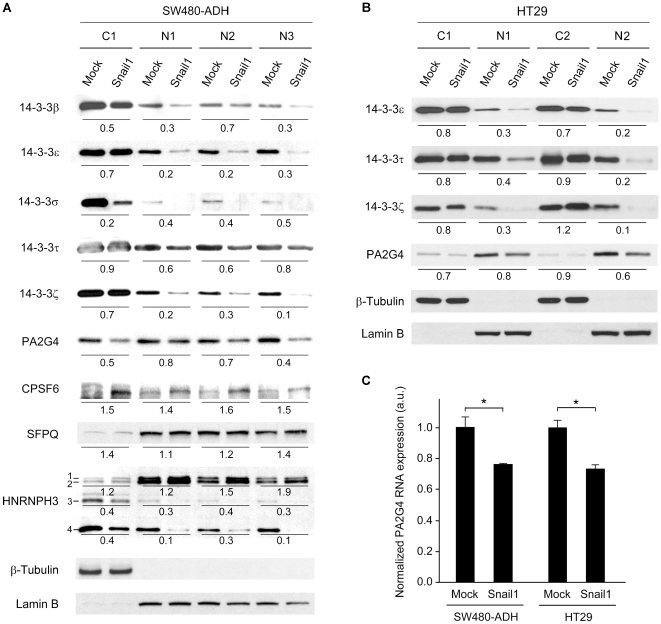
Validation of candidate Snail1 target proteins. (A) Western blot analysis of the expression of selected proteins in cytosolic (C) and nuclear (N) extracts of SW480-ADH Mock and Snail1 cells. HNRNPH3 isoforms (1–4) are indicated. The numbers below the gels indicate the quantification of the change in expression between Snail1 and Mock cells after normalization to β-Tubulin or Lamin B (cytosolic and nuclear control, respectively). The statistical significance of the nuclear expression changes promoted by Snail1 was assessed by two-tailed unpaired Student's *t*-test. It was *p*<0.001 for 14-3-3ε, 14-3-3σ, 14-3-3ζ, CPSF6 and the isoforms 3 and 4 of HNRNPH3; *p*<0.01 for 14-3-3τ; and *p*<0.05 for 14-3-3β and PA2G4. The regulation of SFPQ (*p* = 0.057) and that of the isoforms 1+2 of HNRNPH3 (*p* = 0.058) did not reached the statistical significance, but a clear tendency was observed. (B) Western blot analysis of the expression of selected proteins in cytosolic (C) and nuclear (N) fractions of HT29 Mock and Snail1 cells. The numbers below the gels indicate the quantification of the change in expression between Snail1 and Mock cells after normalization to β-Tubulin or Lamin B (cytosolic and nuclear control, respectively). (C) Quantitative RT-PCR study of PA2G4 expression in SW480-ADH and HT29 Mock and Snail1 cells. 18S rRNA expression was used for normalization. Statistical significance was assessed by two-tailed unpaired Student's *t*-test, single asterisk indicates *p*<0.05.

For a quantitative estimation of the expression of the candidate target proteins in SW480-ADH Mock and Snail1 cells, we analyzed by Western blot three nuclear extracts prepared independently (N1, N2, N3). Additionally, the cytosolic extracts from one of the experiments (C1) were included in the analysis (Figure 3A). Variable downregulation (20% to 70%) of 14-3-3 β, ε, σ, τ and ζ proteins were found in the nucleus of Snail1 cells. For all of them except 14-3-3σ the downregulation was weaker in the cytosol than in the nucleus (Figure 3A). We have been unable to detect 14-3-3η expression neither in the nucleus nor in the cytosol, whereas that of 14-3-3γ was unaffected by Snail1 overexpression (data not shown). PA2G4 expression was also lower in both nucleus and cytosol of SW480-ADH Snail1 than in Mock cells (Figure 3A).

By contrast, the level of CPSF6 was higher in Snail1 cells than in Mock cells (Figure 3A). We have also analyzed the expression of Splicing factor proline/glutamine-rich (SFPQ), a protein found 1.59-fold upregulated in the proteomic analysis, but absent from Table 1 because it was identified only with one peptide in spot 609. We did so because both SFPQ and CPSF6 are involved in pre-mRNA processing and localized in nuclear paraspeckles [23], which suggests a possible coordinated effect of Snail1. Indeed, we found that Snail1 increased nuclear SFPQ expression and also the low level of SFPQ detected in the cytosol (Figure 3A).

The regulation by Snail1 was complex in the case of the Heterogeneous nuclear ribonucleoprotein H3 (HNRNPH3), which presents several isoforms resulting from alternative splicing [24]. The peptides used in the proteomic study to identify HNRNPH3 indicate that the downregulated spot may only correspond to isoforms 1, 2 or 3. The analysis by Western blot showed that the expression of isoforms 1 and 2 (predicted MW 36.9 and 35.2 kDa, respectively) was upregulated by Snail1, while that of isoforms 3 and 4 (predicted MW 31.5 and 22.3 kDa, respectively) was strongly downregulated in both nucleus and cytosol of Snail1 cells (Figure 3A). Therefore, the protein identified in the proteomic analysis must be isoform 3 of HNRNPH3.

The repression of 14-3-3ε, 14-3-3τ, 14-3-3ζ and PA2G4 by Snail1 in nuclear extracts was reproduced in two independent experiments in another human colon cancer cell line, HT29 (Figure 3B). In addition, quantitative RT-PCR analysis revealed a decreased level of PA2G4 RNA in both SW480-ADH and HT29 cells expressing Snail1 as compared to their respective Mock cells (Figure 3C).

### Subcellular distribution of Snail1 target proteins

**Figure 4:**
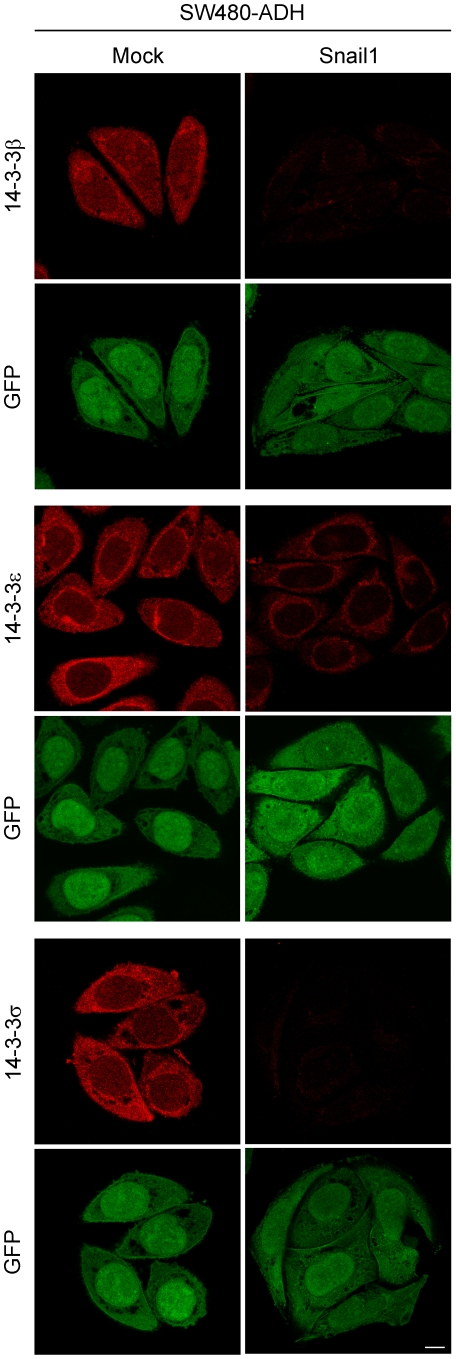
Immunofluorescence analysis of 14-3-3 proteins in Snail1 and Mock cells. Representative confocal microscopy images showing the expression and localization of 14-3-3 β, ε and σ (red) in SW480-ADH Mock and Snail1 cells. GFP expression (green) was unaltered by Snail1 and was used as a control. Scale bar, 10 µm.

**Figure 5:**
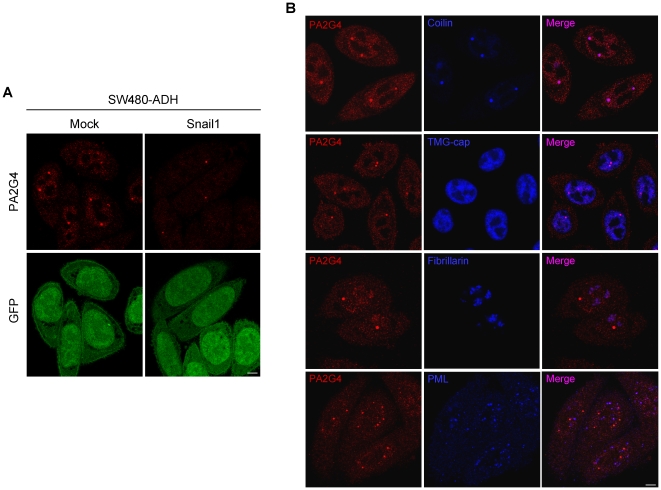
Subcellular distribution of PA2G4. (A) Representative confocal microscopy images showing the expression and localization of PA2G4 (red) in SW480-ADH Mock and Snail1 cells. GFP expression (green) was unaltered by Snail1 and was used as a control. Scale bar, 5 µm. (B) Representative confocal microscopy images showing double immunolabeling for PA2G4 (red) and for molecular markers (blue) of Cajal bodies (Coilin), Cajal bodies and nuclear speckles of splicing factors (TMG-cap), nucleolus (Fibrillarin), and PML bodies (PML) in SW480-ADH Mock cells. The pink colour in the merge images indicates the existence of colocalization. Scale bar, 5 µm.

The expression of some of the Snail1 target proteins validated by Western blot was further studied by immunofluorescence and confocal microscopy analyses. As shown in Figure 4, the level of 14-3-3 β, ε and σ was much lower in both nucleus and cytosol of SW480-ADH Snail1 cells than in Mock cells. In all three cases the expression was higher in the cytosol than in the nucleus with a variable ratio: β<σ<ε. Immunofluorescence analysis also showed a reduction of both nuclear and cytosolic expression of PA2G4 in Snail1 cells compared to Mock cells (Figure 5A). The expression of PA2G4 had a fine punctate pattern in both nucleus and cytosol, and was particularly strong in a few round and sharply defined nuclear foci of approximately 0.5 µm in diameter (Figure 5). Double staining using antibodies against Coilin (marker of Cajal bodies), the TMG-cap of spliceosomal snRNAs (marker of Cajal bodies and nuclear speckles of splicing factors), Fibrillarin (marker of nucleolus) and PML (marker of PML bodies) indicated that the PA2G4-positive nuclear foci correspond to Cajal bodies (Figure 5B). This result was confirmed in neurons (data not shown), the cell type where the Cajal bodies are most prominent [25]. The best understood function of Cajal bodies is the biogenesis of small nuclear and nucleolar RNPs (snRNPs, snoRNPs) involved in pre-mRNA splicing and pre-rRNA processing, respectively [25], [26]. The double staining experiments also showed a faint staining of PA2G4 in the nucleolus (see Figure 5B, PA2G4 and Fibrillarin co-staining), as it has been previously described [27].

### Analysis of human colon tumors

**Figure 6:**
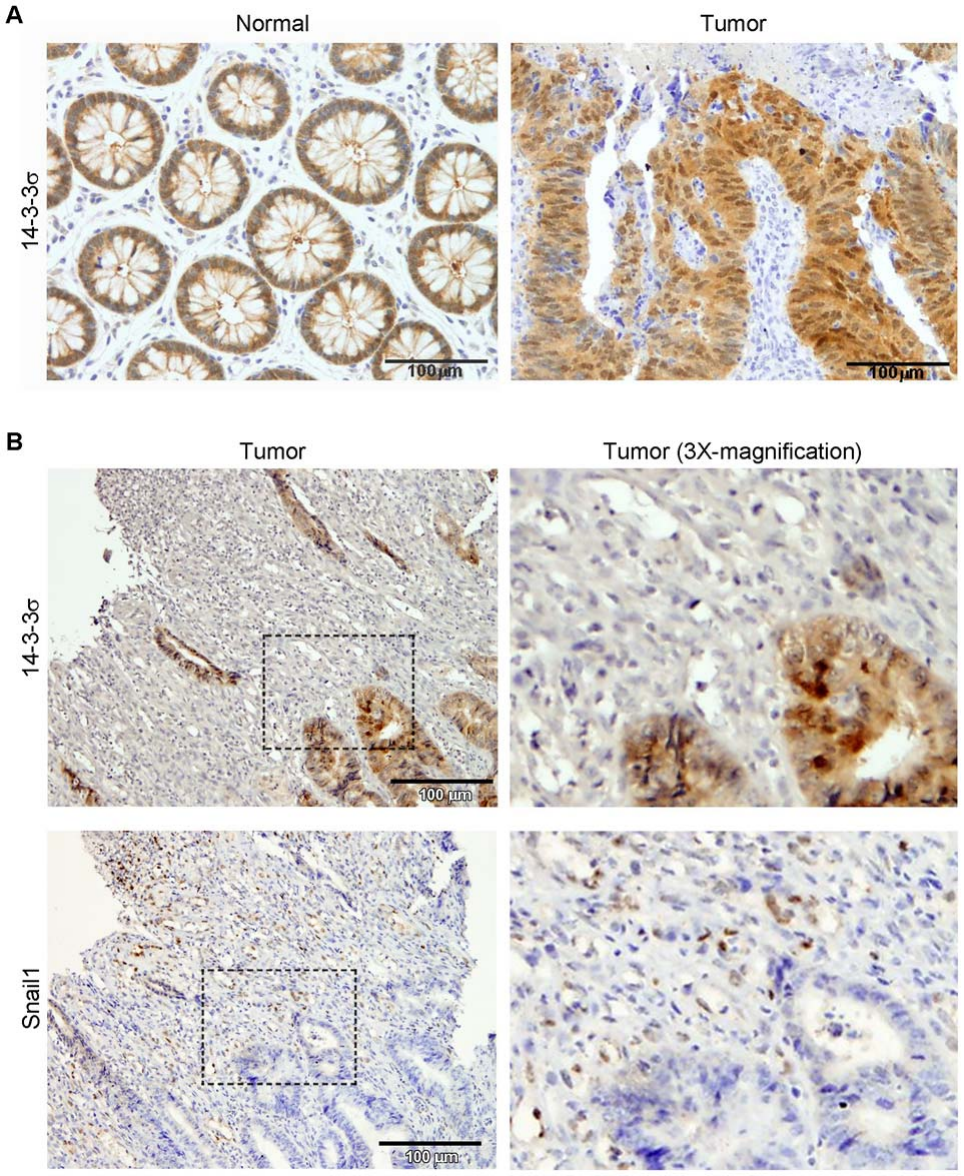
Immunohistochemistry staining of 14-3-3σ and Snail1 in human colon tumors. (A) Immunohistochemistry images showing 14-3-3σ expression in representative normal and tumor colon samples of the 46 analyzed in the tissue array. Bars indicate magnification. Sections were counterstained with haematoxylin. (B) Immunohistochemistry images showing 14-3-3σ and Snail1 expression in two consecutive slices of a representative colon cancer tumor of the five analyzed. Bars indicate magnification. Sections were counterstained with haematoxylin. Right panels are a 3X-magnification of the region indicated in left panels.

The repression of several 14-3-3 family members by a tumor-associated transcription factor as Snail1 prompted us to study the expression of these proteins in human colon cancer. As 14-3-3σ was the member which expression was more strongly repressed by Snail1 both in the nucleus and in the cytosol, we selected it for the study on human samples. First, we analyzed 14-3-3σ expression by immunohistochemistry in a tissue array containing paired normal and tumor samples from 46 colorectal cancer patients, and found that 14-3-3σ was highly expressed by both normal and tumor epithelial cells but not by stromal cells (Figure 6A). As this expression pattern of 14-3-3σ was coherent with its repression by an inducer of EMT such us Snail1, we decided to perform a more detailed study of 14-3-3σ and Snail1 expression in consecutive slices of five colon tumor biopsies. In agreement with previous studies [13], we mainly found Snail1 immunoreactivity in the nucleus of cells with mesenchymal phenotype within the tumor (Figure 6B). These cells could correspond to tumor epithelial cells that have undergone EMT and/or fibroblasts that have been activated by the tumor microenvironment [13]. Accordingly with the data obtained from the tissue array, 14-3-3σ was expressed in both nucleus and cytosol of tumor epithelial cells, but it was absent in stromal cells (Figure 6B). Therefore, 14-3-3σ and Snail1 have opposite expression patterns (epithelial and stromal, respectively) in colon cancer. In addition, 14-3-3σ expression closely resembles that of the Snail1 target gene *CDH1*/E-cadherin and, as proposed for this gene, it is possible that Snail1 represses 14-3-3σ expression in mesenchymal cells.

## Discussion

Identifying proteins regulated by Snail1 widens the present knowledge on the biology of this transcription factor and sheds new light on the mechanisms of EMT and cancer progression. We chose colon cancer as a working system because Snail1 is upregulated at both RNA and protein level in this neoplasia and contributes to its malignization [10]–[13]. Previously, the gene expression profiles induced by Snail1 in different systems had been studied by transcriptomic analyses, and most of the Snail1 target genes identified encoded for proteins involved in cellular adhesion, extracellular matrix and cytoskeleton [4], [15], [28]. In this study, we performed a proteomic analysis of nuclear extracts aimed to identify novel proteins regulated by Snail1 that could mediate its wide effects on cell physiology and phenotype.

Our study shows a remarkable conservation in the global pattern of nuclear protein expression in human colon cancer cells that express high or low levels of Snail1. Still, a series of previously uncharacterized Snail1-regulated proteins implicated in diverse cell functions have been identified. Twenty-two spots were found as significantly deregulated between both cell types with a *p*<0.05 (Student's *t*-test). Nineteen different proteins were identified, most of them by using the ESI-linear ion trap instrument in combination with a nano-HPLC. Only five proteins were identified by MALDI-TOF-TOF, probably due to the low expression of the proteins present in the spots. Two spots (1419 and 1926) contained more than one protein. However, in spot 1419, Vimentin was significantly more abundant (based on the number of identified peptides) than the other proteins and we assigned to it the differences in expression. In spot 1926, the number of identified peptides was quite similar between LASP1 and HNRNPH3, making more difficult the assignation of the differential expression.

Our results agree with the transcriptional repressor role of Snail1, as in 12 out of the 17 spots with identified proteins the expression was lower in Snail1-expressing cells than in Mock cells, while only in five of them the expression was higher in Snail1 cells. The repression of the downregulated proteins could be a direct effect of Snail1, especially in the case of PA2G4 whose RNA level was also reduced by Snail1. By contrast, the induction of protein expression by Snail1 must be indirect, mediated by other transcription factors, as it has been reported for Matrix metalloproteases 2 and 9 [29], [30]. In addition, we found that Snail1 alters the splicing pattern of HNRNPH3 in SW480-ADH cells. This is in line with studies describing that Snail1 changes the pattern of splicing of p120-Catenin and *Zonula occludens*-1 by uncharacterized mechanisms [31], [32].

In agreement with previous transcriptomic studies, we show here that Snail1 changes the expression of proteins involved in cell adhesion and cytoskeleton (CTNNA1, CALD1, VIM and LASP1). Additionally and to our knowledge for the first time, we report that Snail1 regulates proteins involved in other cellular functions as RNA maturation (CPSF6, HNRNPH3 and SFPQ) and intracellular signaling (PI3KR1, RANBP1 and 14-3-3 protein family). The almost general repressive effect of Snail1 on the expression of 14-3-3 family members is surprising and agrees with the recently proposed cancer-protecting role of certain of these proteins [33], [34]. The 14-3-3 proteins have for long been known as phosphoserine/phosphothreonine binding proteins able to bind and modulate the interaction between proteins and involved in cell processes as signaling, cell cycle control, apoptosis, intracellular trafficking, cytoskeletal structure and transcription [34], [35]. Only in recent years several 14-3-3 family members have been linked to a variety of cancer processes. While 14-3-3σ has been proposed as a tumor suppressor gene that is silenced or inactivated during progression of melanoma and breast, gastric, hepatocellular, ovarian and squamous cell carcinomas [33], [36]–[40]; it has been found overexpressed in pancreatic cancer [41]. In addition, a proteomic analysis showed that 14-3-3σ expression is downregulated in invasive transitional cell carcinomas of the urinary bladder undergoing EMT [19]. This is in agreement with our data from human colon cancer biopsies that show an opposite expression pattern of 14-3-3σ and Snail1. Notably, 14-3-3 ε, ζ and η can form a ternary complex with Chibby and β-Catenin facilitating nuclear export of β-Catenin, thereby antagonizing its transcriptional activity that is an important driver in colon cancer [42]. Contrarily, 14-3-3ζ cooperates with ErbB2 to promote EMT and progression of ductal breast carcinomas to invasive cancer [43], and its overexpression associates with breast cancer recurrence [44]. In this study, we have identified 14-3-3 β, ε, σ, τ and ζ as downregulated after Snail1 expression in human colon cancer cells. The action of the 14-3-3 proteins can be modulated by interaction with different partners in a spatially and temporally fashion and also is subjected to regulation by phosphorylation. Therefore, the same 14-3-3 family member can have multiple and even opposing roles making the analysis of the effect of individual 14-3-3 proteins very complex and dependent on the integration of additional signals [45]. Therefore, it is difficult to predict the role of these proteins in Snail1 action in colon cancer, which may be related to the mutations typically present in this neoplasia.

We found that Snail1 represses also PA2G4 expression in human colon cancer cells. This protein interacts with the cytoplasmic domain of ErbB3 receptor downregulating ErbB signal transduction and attenuating heregulin-driven growth of breast cancer cells [46], [47]. PA2G4 is located not only in the cytosol but also in the nucleus where it behaves as a pleiotropic protein that interacts with a number of proteins and RNAs involved in transcriptional regulation and translation control [48], [49]. Thus, PA2G4 inhibits E2F1- and androgen receptor-mediated transcription through its interaction with retinoblastoma protein, androgen receptor, several histones deacetylases and Sin3A [50]–[53]. The localization of PA2G4 in Cajal bodies that we have now reported suggests that this multifunctional protein is also involved in the maturation of snRNA and snoRNA. Moreover, PA2G4 overexpression induces differentiation of human breast cancer cells [54], inhibits proliferation of human fibroblasts, and suppresses growth and metastasis of salivary adenoid cystic carcinoma cells [55]. From all these studies it is conceivable that PA2G4 repression may contribute to the tumorigenic action of Snail1.

Interestingly, Snail1 upregulates the expression of CPSF6 and SFPQ proteins involved in RNA polyadenylation and splicing, respectively [56], [57]. Both proteins have been recently found to be present in paraspeckles, a subnuclear compartment proposed to be site for pre-mRNA processing and retention [23]. Of note, both CPSF6 and SFPQ are fusion partners for tyrosine kinases in haematological malignancies [58]. Snail1 also alters the splicing pattern of HNRNPH3, another protein involved in the splicing process [24]. Intriguingly, the HNRNPH3 isoforms induced by Snail1 contain two copies of a RNA-binding domain (RNA recognition motif) commonly found in proteins that bind single-stranded RNA, while those isoforms repressed by Snail1 contain only one [24]. However, the functional consequence of this difference is unknown. Therefore, our study indicates that Snail1 may modulate several steps in RNA maturation, such as polyadenylation and splicing, and so mRNA stability and translation. Altogether, these data suggest that in addition to its recognized transcription repression activity, Snail1 may regulate gene expression post-transcriptionally by modulating the level of proteins involved in pre-mRNA processing and location.

In conclusion, our findings implicate Snail1 as a regulator of previously unpredicted cellular functions in addition to intercellular adhesion and cell morphology. The in-depth study of the mechanisms of action of the newly identified Snail1 target proteins will contribute to define the precise complex biological activity of Snail1 and so the insights of the process of EMT during embryogenesis and tumorigenesis.

## Materials and Methods

### Ethics statement

This study was conducted according to the principles expressed in the Declaration of Helsinki. The study was approved by the Ethical Committee of the Instituto de Salud Carlos III (Madrid, Spain) and the Ethical Committee for Clinical Research of the Institut Municipal d'Assistència Sanitària (Barcelona, Spain). All patients provided written informed consent for the collection of samples and subsequent analysis.

### Cell culture

SW480-ADH and HT29 human colon cancer cells stably expressing Snail1 (Snail1 cells) or an empty vector (Mock cells) were generated by retroviral transduction using pRV-Snail1-IRES-GFP (Snail1 cells) or pRV-IRES-GFP (Mock cells) vectors as we have previously reported [10]. Cells were grown in DMEM supplemented with 10% FCS and 1 mM glutamine (all from Invitrogen, Carlsbad, CA). Phase-contrast images were captured with a DC300 digital camera (Leica Microsystems, Wetzlar, Germany) mounted on an inverted Leitz Labovert FS microscope.

### Subcellular fractioning

To obtain subcellular fractions, cell monolayers were washed in PBS and lysed for 15 min in 500 µl hypotonic buffer (10 mM HEPES pH 7.5, 10 mM KCl, 0.1 mM EDTA, 0.1 mM EGTA) supplemented with 1 mM DDT, 1 mM ortovanadate, 1 mM PMSF, 10 µg/ml leupeptin and 10 µg/ml aprotinin. Just before centrifugation (13,000 rpm at 4°C for 10 min), 31.5 µl of 10% NP40 were added to the tubes. Supernatants containing the cytosolic extracts were maintained at −80°C until analysis. Pellets containing nuclei were lysed by incubation for 30 min in hypertonic buffer (20 mM HEPES pH 7.5, 0.4 M NaCl, 1 mM EDTA, 1 mM EGTA) supplemented with 1 mM DDT, 1 mM ortovanadate, 1 mM PMSF, 10 µg/ml leupeptin and 10 µg/ml aprotinin, and then centrifuged 13,000 rpm at 4°C for 10 min. Supernatants (nuclear extracts) were conserved at −80°C.

### 2D-DIGE electrophoresis

Nuclear extracts were precipitated using the 2D Clean-UP kit (GE Healthcare, Waukesha, WI) and resuspended in the appropriate 2D-DIGE lysis buffer (7 M urea, 2 M thiourea, 25 mM Tris-HCl pH 8.5, 4% CHAPS) [59]. Fifty µg of each nuclear protein extract were labeled with 400 pmol CyDyes on ice for 30 min in the dark as previously described [59], [60]. Nuclear protein extracts from SW480-ADH Mock and Snail1 cells were labeled with Cy3 and Cy5 fluorochromes, alternating the dyes to avoid labeling bias. A pool was generated by combining an equal amount of extract from each cell type. This pool was labeled with Cy2 dye and was included in all gel runs. Samples were run using commercial IPG strips for the IEF (pH 3–10 NL, 24 cm length) and standard continuous 12% SDS-PAGE for the second dimension. Finally, a preparative gel containing 500 µg of each nuclear extract was run and stained with Sypro Ruby (Invitrogen) for protein visualization and spot picking after matching against the analytical gels.

Proteins were visualized with the fluorescence scanner Typhoon 9400 (GE Healthcare). The images were processed using DeCyder software v.6.5. The DeCyder differential in-gel analysis (DIA) module was used for pair-wise comparisons of SW480-ADH Mock and Snail1 cells to the mixed standard present in each gel and for the calculation of normalized spot volumes/protein abundance. The matching between gels was performed using the internal pool included in each gel. The 12 spot maps corresponding to four replica gels were used to calculate average abundance changes and paired Student's *t*-test *p*-values for each protein across the different gels. To this end, we used the DeCyder biological variation analysis (BVA) module and the Cy3∶Cy2 and Cy5∶Cy2 ratios for each individual protein. Protein spots that showed a significant (Student's *t*-test, *p*<0.05) change in abundance between the two cell types were selected for further characterization by mass spectrometry.

### Identification of proteins by MALDI-TOF-TOF peptide mass fingerprinting

Spots of interest were excised from the gel automatically using an Ettan-Picker robot (GE Healthcare) and subjected to tryptic digestion. Proteins were first reduced (10 mM DTT) and then alkylated (50 mM iodoacetic acid). Following vacuum-drying, the gel pieces were incubated with 10 ng/ µl of modified porcine trypsin (Promega, Madison, WI) in 50 mM ammonium bicarbonate for 16 h at 37°C. Supernatants were collected, vacuum-dried, re-dissolved in 0.5 µl 0.1% trifluoroacetic acid and added onto a matrix consisting of 0.5 µl of 5 mg/ml α-ciano-hydroxycynamic in water∶acetonitrile (2∶1) with 0.1% trifluoroacetic acid. MALDI-TOF-TOF analysis of the samples was carried out on a 4800 Instrument (Applied Biosystems, Framingham, MA) in positive ion reflector mode. Following MS analysis, the instrument was switched to MS/MS mode, and the five strongest peptides from the MS scan were isolated and fragmented by collision-induced dissociation. The ion acceleration voltage was 20 kV. The obtained MALDI-MS data were further processed using the GPS Explorer Software (Applied Biosystems) that acts as an interphase between the Oracle database containing the raw spectra and a local copy of the MASCOT search engine. The *Homo sapiens* subset of the sequences in the SwissProt and Celera databases were utilized for searches using MASCOT search engine. Carboxymethylation (Cys) and oxidation (Met) as fixed and variable modifications, respectively, were taken into account for database searching. The following search parameters were used in all MASCOT searches: tolerance of one missed cleavage, methionine oxidation (+16 Da), and a maximum error tolerance of 100 ppm in the MS data and 0.8 Da in the MS/MS data.

### Identification of proteins by peptide sequencing using ESI-linear ion trap

As MALDI-TOF-TOF analysis failed to identify proteins on several spots, tryptic peptides were analyzed by nano-HPLC-MS/MS using an ESI-linear ion trap mass spectrometer (Thermo Fisher Scientific, Waltham, MA) according to previous protocols [61]. Peptides were eluted at a 300 nl/min constant flow rate on a reverse phase PepMap C18 column (75 µm id ×15 cm) (Dionex, Sunnyvale, CA) using a 60 min linear gradient from 4 to 60% solvent B, being solvent A 0.1% formic acid in water and solvent B 0.1% formic acid in acetonitrile. All HPLC runs were performed using an Ultimate 3000 nano-HPLC system (Dionex) operated at a 300 nl/min constant flow rate. The peptides were scanned and fragmented with a linear ion trap mass spectrometer (Thermo Fisher Scientific) equipped with a dynamic nano-ESI source (Proxeon, Odense, Denmark). An electrospray voltage of 1700 V and a capillary voltage of 50 V at 190°C were set up. Fragmentation spectra were searched against SwissProt v.57.4 database using SEQUEST as search engine. The following parameters were used for searches using SEQUEST v.3.3.1 SP1: enzyme, trypsin; fixed modifications, carboxymethyl cysteine; variable modifications, oxidation of methionine; peptide tolerance, ±1.50 amu; fragment ion tolerance, ±0.5 amu; and number of missed cleavage sites, 1. All the proteins identified were reanalyzed using a≤1% false positive discovery rate (FDR) cut-off by searching against a decoy database (a reverse-concatenated randomized database) using the Proteome Discoverer software (Thermo Fisher Scientific). Protein location and biological functions were assigned using the AmiGO software (release date 10/07/09).

### Western blotting

To obtain whole-cell extracts, cells were lysed in SDS buffer (25 mM Tris-HCl pH 7.6, 1 mM EDTA, 1 mM EGTA and 2% SDS) supplemented with 1 mM ortovanadate, 1 mM PMSF, 10 µg/ml leupeptin and 10 µlg/ml aprotinin. Lysates were sonicated in a Bioruptor (Diagenode, Liège, Belgium), centrifuged at 13,000 rpm for 10 min at 4°C and supernatants were conserved at −80°C. Whole-cell and subcellular extracts were separated by SDS-PAGE electrophoresis and transferred to Immobilon P membranes (Millipore, Billerica, MA). The membranes were incubated with the appropriate primary and secondary horseradish peroxidase-conjugated antibodies, and the antibody binding was visualized using the ECL detection system (GE Healthcare). Quantification was done by densitometry using ImageJ software. The expression of the proteins of interest was then normalized to that of β-Tubulin (for citosolic extracts) or Lamin B (for nuclear extracts), and statistical significance was assessed by two-tailed unpaired Student's *t*-test in the indicated cases.

The following primary antibodies were used: 14-3-3β, 14-3-3ε, 14-3-3τ, 14-3-3ζ and Lamin B (sc-25276, sc-23957, sc-732, sc-1019 and sc-6216, Santa Cruz Biotechnology, Santa Cruz, CA); 14-3-3σ (012202F, Thermo Fisher Scientific); SFPQ and CPSF6 (H00006421-M02 and H00011052-M10, Abnova Corporation, Taipei, Taiwan); PA2G4 (07-397, Millipore); HNRNPH3 (ARP40721_T100, Aviva Systems Biology, San Diego, CA); HA (MMS-101R, Covance, Princeton, NJ); E-cadherin (610181, BD Biosciences, San Jose, CA); Occludin and Claudin-7 (71-1500 and 34-9100, Invitrogen); Lef-1 (2286, Cell Signaling, Danvers, MA); and β-Tubulin (T4026, Sigma-Aldrich, St Louis, MO).

### Immunofluorescence and confocal microscopy

Cells were rinsed once in PBS, fixed in 3.7% paraformaldehyde for 15 min at RT and rinsed once in 0.1 M glycine and twice in PBS. The cells were permeabilized in 0.5% Triton X-100 and then washed three times in PBS. Nonspecific sites were blocked by incubation with PBS containing 2% BSA for 30 min at RT before incubating the cells with the primary antibodies diluted 1/100 in PBS O/N at 4°C. After four washes in PBS, the cells were incubated with secondary fluorophore-conjugated antibodies for 45 min at RT, washed and mounted in VectaShield (Vector Laboratories, Burlingame, CA). For F-actin staining, cells were incubated with TRITC-conjugated phalloidin (Sigma-Aldrich) for 15 min at RT and washed in PBS. Confocal microscopy was performed with a Zeiss LSM510 laser scanning microscope (Oberkochen, Germany) and using a 63x oil (1.4 NA) objective. For double labelling experiments, images of the same confocal plane were sequentially recorded by sequential excitation at 488/543/633 nm to detect FITC, Cy3 and Cy5, respectively.

We used primary antibodies against: 14-3-3β, 14-3-3σ and PML (sc-25276, sc-100638 and sc-966, Santa Cruz Biotechnology); 14-3-3ε (610542, BD Biosciences); PA2G4 (07-397, Millipore); Coilin (ab11822, Abcam, Cambridge, UK); 2,2,7-trimethylguanosine cap (TMG-cap) of spliceosomal small nuclear RNAs (snRNAs) (NA02, Oncogene Research, San Diego, CA); and Fibrillarin (72B9) [62].

### RNA extraction and quantitative RT-PCR

RNA was extracted from cultured cells using RNeasy Mini Kit (Qiagen, Hilden, Germany). The level of PA2G4 RNA was measured by quantitative RT-PCR in relation to that of 18S rRNA using the comparative C_T_ method and RNA TaqMan probes (Applied Biosystems). RNA was retrotranscribed using the High-Capacity cDNA Archive Kit (Applied Biosystems). The quantitative PCR reaction was performed in an ABI Prism 7900 HT thermal cycler using TaqMan Gene Expression Master Mix (both from Applied Biosystems). Thermal cycling consisted of a denaturing step at 95°C for 10 min and 40 cycles of denaturing at 95°C for 15 s and annealing and elongation at 60°C for 60 s. Statistical significance was assessed by two-tailed unpaired Student's *t*-test, single asterisk indicates *p*<0.05.

### Immunohistochemistry

Tissue arrays containing normal and tumor paired samples from 46 colorectal cancer patients were prepared as previously described [63]. These samples were obtained from the Tumor Bank of Centro Nacional de Investigaciones Oncológicas (Instituto de Salud Carlos III, Madrid, Spain). The arrays were incubated with 14-3-3σ antibody (diluted 1/50, sc-100638, Santa Cruz Biotechnology) and visualization of specific interactions was monitored using the Envision System (Dako, Glostrup, Denmark). In addition, we studied 14-3-3σ and Snail1 expression using consecutive sections from five human colon tumors obtained from the Tumor Bank of the Department of Pathology of the Hospital del Mar (Institut Municipal d'Assistència Sanitària, Barcelona, Spain). Tissues were sectioned at 4 µm, deparaffined and rehydrated using xylene and a series of graded ethanol. For antigen unmasking, sections were immersed in Tris-EDTA pH 8 buffer (10 mM Tris-HCl pH 8, 1 mM EDTA, 10 mM NaCl), boiled for 5 or 20 min (for 14-3-3σ or Snail1, respectively), cooled at RT for 20 min, and rinsed with PBS. Immunohistochemical staining was carried out with 14-3-3σ antibody (at the same conditions as above) and with Snail1 antibody (diluted 1/300, MAb EC) [64] using the PowerVision Poly-HRP Amplification System (Leica Microsystems) for 14-3-3σ and the CSAII Amplification System (Dako) for Snail1. Sections were counterstained with haematoxylin.
